# Prenatal Arsenic Exposure on DNA Methylation of *C18ORF8* and *ADAMTS9* Genes of Newborns from the POSGRAD Birth Cohort Study

**DOI:** 10.3390/toxics12070476

**Published:** 2024-06-30

**Authors:** Carolina Lerma-Treviño, Leticia Hernández-Cadena, Jorge Octavio Acosta-Montes, Georgina Hernández-Montes, Isabel Alvarado-Cruz, Isabelle Romieu, Albino Barraza-Villarreal

**Affiliations:** 1 Centro de Investigación en Nutrición y Salud, Instituto Nacional de Salud Pública, Cuernavaca 62100, Mexico; carolinalermat@gmail.com; 2 Dirección de Salud Ambiental, Instituto Nacional de Salud Pública, Cuernavaca 62100, Mexico; lhcadena@insp.mx (L.H.-C.); iromieu@gmail.com (I.R.); 3 Facultad de Enfermería y Nutriología, Universidad Autónoma de Chihuahua, Chihuahua 31000, Mexico; jacostanutricion@gmail.com; 4 CIC-UNAM-Instituto Nacional de Ciencias Médicas y Nutrición Salvador Zubirán, Mexico City 14080, Mexico; yinna@cic.unam.mx; 5 Department of Cellular and Molecular Medicine, University of Arizona, Tucson, AZ 85724, USA; ialvarado@arizona.edu

**Keywords:** DNA methylation, development programming, *C18ORF8*, MIC-1, Arsenic, DHA

## Abstract

Exposure to arsenic (As) is a public health problem associated with cancer (skin and colon) and it has been reported that epigenetic changes may be a potential mechanism of As carcinogenesis. It is pertinent to evaluate this process in genes that have been associated with cancer, such as *ADAMTS9* and *C18ORF8.* Gestation and delivery data were obtained from the POSGRAD study. Exposure to As was measured in urine during pregnancy. Gene methylation was performed by sodium bisulfite sequencing; 26 CpG sites for the *C18ORF8* gene and 21 for *ADAMTS9* were analyzed. These sites are located on the CpG islands near the start of transcription. Sociodemographic characteristics were obtained by a questionnaire. The statistical analysis was performed using multiple linear regression models adjusted for potential confounders. Newborns with an As exposure above 49.4 μg g^−1^ showed a decrease of 0.21% on the methylation rate in the sites CpG15, CpG19, and CpG21 of the *C18ORF8* gene (adjusted ß = −0.21, *p*-value = 0.02). No statistically significant association was found between prenatal exposure to As and methylation of the *ADAMTS9* gene. Prenatal exposure to As was associated with decreased DNA methylation at the CpG15, CpG19, and CpG21 sites of the *C18ORF8* gene. These sites can provide information to elucidate epigenetic mechanisms associated with prenatal exposure to As and cancer.

## 1. Introduction

Environmental exposure to arsenic (As) is still one of the main global public health concerns. It has been estimated that more than 200 million people are exposed to concentrations above 10 μg L^−1^, the reference level recommended by the World Organization of Health (WHO) [1]. Mexico is one of the most affected countries, estimating that approximately six million inhabitants have an exposure to As especially naturally [2]. Similarly, the state of Morelos is considered an area with significant concentrations of As, primarily due to mining activities [3,4,5].

Arsenic is one of the most toxic metals and is highly carcinogenic, which is one of the most relevant and preoccupying health effects for public health research [6]. Among the malignant tumors linked to exposure to As, bladder, lung, kidney, liver, skin, and colon tumors can be found [7,8,9]. The latter is one of the cancers that cause the most deaths in the world [10]. Each year, more than 240,000 new cases of colon cancer are reported throughout America and approximately 112,000 deaths are estimated from this disease [11].

Epigenetic changes have been considered as one of the potential pathological mechanisms to explain the carcinogenesis of As [12] as it has been shown that As alters gene expression through epigenetic mechanisms. Therefore, it is proposed that these alterations in gene expression could promote phenotypes that do not control deterioration and resistance to apoptosis [13,14].

Alteration of DNA methylation is a relevant epigenetic mechanism that regulates gene expression, mainly in regions with a high concentration of phosphate-linked cytosine and guanine pairs, called CpG islands [15], close to the beginning of gene transcription [16,17]. DNA methylation has two effects which have been associated with the carcinogenesis process. On the one hand, hypomethylation or a decrease in 5-methylcytosine (5-mC) marks at CpG sites, which is generally associated with the gene expression of specific proteins involved in cancer invasion and metastasis, and methylation or a gain of 5-mC that results in gene silencing, such as tumor suppressor genes [18].

The effects of exposure to environmental pollutants on DNA methylation have been previously reported and have been associated with both increases and decreases in methylation in chronically exposed adults and infants exposed during pregnancy [19]. In this sense, a study conducted in Mexico identified [2,9,19] genes with differences in methylation associated with prenatal exposure to arsenic [20]. In another study conducted in Mexico, they evaluated DNA methylation in umbilical cord blood and reported a decrease in methylation levels of the 8-oxoguanine DNA glycosylase gene (OGG1) associated with prenatal exposure to As (*p* = 0.01) [21]. This gene codifies an enzyme responsible for the cleavage of 8-oxoguanine, a common oxidative lesion in DNA [22]. A group of researchers in Bangladesh evaluated prenatal exposure to As and its association with DNA methylation [12], finding higher methylation in some of the CpG sites of the p16 gene, which is involved in cell regulation, and also codifies a tumor suppressor protein [23].

The evaluation of epigenetic processes allows us to identify information on the genes silenced or expressed in cancerous tumors [24]. The genes analyzed in this study, Chromosome 18 open reading frame 8 protein (*C18ORF8/RMC1*) and a disintegrin and metalloproteinase with thrombospondin motifs 9 (*ADAMTS9*) have been highlighted for their association with colon cancer tumors [25,26]. Similarly, they were selected because there is evidence of Single Nucleotide Polymorphisms (SNPs) in these genes [26,27] and they have not been explored in the Mexican population exposed to As. The *C18ORF8* gene encodes the protein MIC-1/GDF15 (Cytokine-1 macrophage inhibitor) which has frequently been reported to be present in this type of neoplasm [28]. The *ADAMTS9* gene encodes a member of the *ADAMTS* family of proteins which are involved in the control of organogenesis during the development and inhibition of angiogenesis [26] in studies conducted on colon cancer. It has been mentioned that the *ADAMTS9* gene may have a role in the suppression of tumors [26,29]; hence, the importance of deepening this kind of study. Thus, the present research was performed to elucidate the association between prenatal exposure to As and methylation of the *C18ORF8* and *ADAMTS9* genes in umbilical cord lymphocyte samples of newborn participants in the POSGRAD cohort study.

## 2. Materials and Methods

### 2.1. Study Design and Population

**Figure 1:**
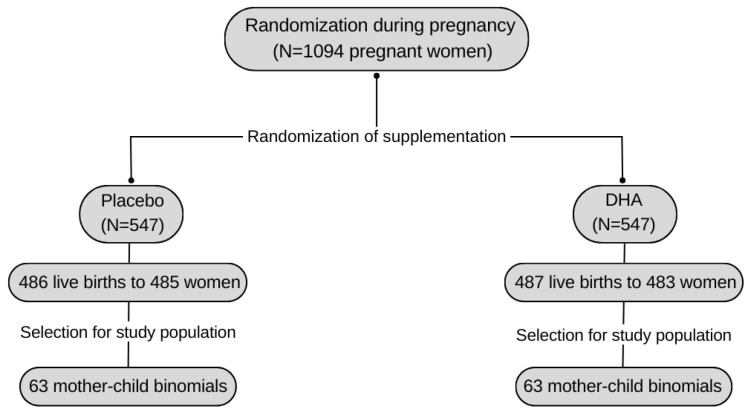
POSGRAD study cohort flow chart showing the selection of the study population.

This research is derived from the POSGRAD birth cohort where 1094 pregnant women were included to evaluate the effect of omega-3 fatty acid (DHA) supplementation on child growth and development. The POSGRAD study began as a double-blind Randomized Clinical Trial during the period of 2005–2009 in Cuernavaca, Morelos, Mexico and for the present report, a subsample of 126 mother-child pairs was selected. For the final analysis, we included only participants which presented complete information of the variables of interest. The flow chart of the sample included is presented in Figure 1. Women were considered for inclusion in the study if they were between 18 and 35 years old, in-between weeks 18 to 22 of gestation and with intentions to have their delivery at the IMSS General Hospital facilities. Women with a high-risk pregnancy, with a history of drug use, metabolic disorders, regular consumption of fish and chronic use of medications for certain diseases were excluded. Details of the methodology have already been specified in previous reports [30].

This study was approved by the Ethics and Biosafety Committee of the National Institute of Public Health of Mexico (Approved number 730 and Project number 418). All participants signed the informed consent for this study.

### 2.2. Data Collection

#### 2.2.1. Analysis of the Methylation of the *C18ORF8* and *ADAMTS9* Genes

**Figure 2:**
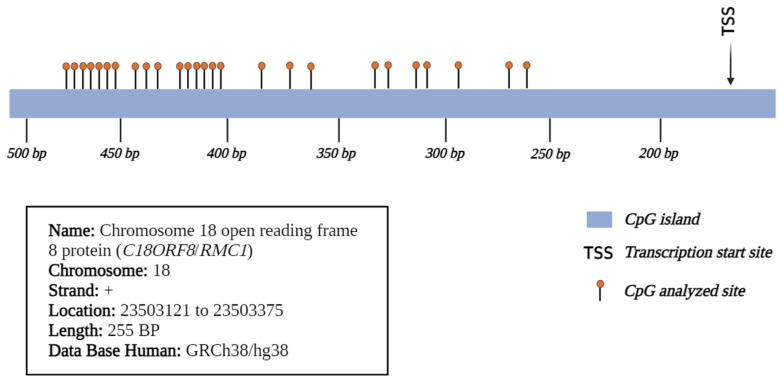
Cytosine-guanine dinucleotide (CpG) sites on the promoter region of the *C18ORF8* gene.

**Figure 3:**
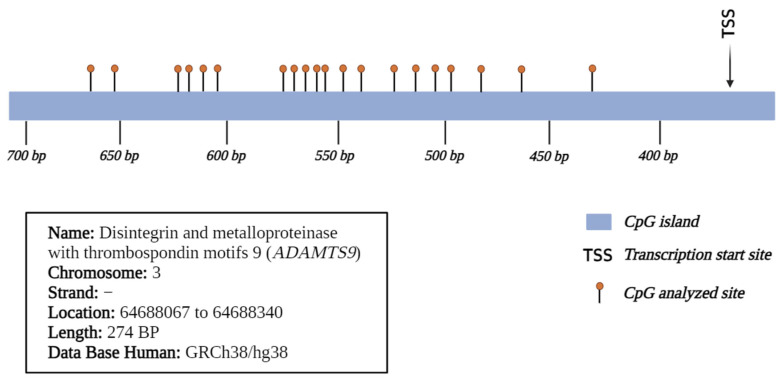
Cytosine-guanine dinucleotide (CpG) sites on the promoter region of the *ADAMTS9* gene.

Cord blood samples were collected from newborns of mothers participating in the clinical trial at the time of delivery. Samples were collected by venipuncture from cord vessels after clamping and cutting the cord and were stored in tubes with EDTA at room temperature for further analysis. The isolate of cord blood mononuclear cells (CBMNC) was realized in the National Institute of Public Health laboratory and the isolation procedure was completed within 12 h after collection. Cord blood was layered over Lymphoprep (Axis-Shield, Dundee, UK). The CBMNC were separated by density centrifugation and stored in a cryogenic storage tank at −80 °C for further analysis and, after the CBMNC, was stored in nitrogen liquid at −250 °C. After the DNA extraction, which was performed with affinity columns (Qiagen), the DNA was treated with bisulfite (EZ DNA Methylation-Gold Kits|ZYMO RESEARCH, México City, México). Subsequently, the sequences of interest were amplified with Amplitaq Gold Master mix (Applied Biosystems) and the reaction mixture included first sense, first anti-sense, and DNA previously treated with bisulfite. Amplification was performed over 30 cycles, and the alignment temperatures for the *C18ORF8* gene sequence were 58 °C and 60 °C for the *ADAMTS9* gene. Subsequently, the amplicon was purified, and the fragments underwent a second PCR reaction for the addition of adapter sequences. The integrity of the amplicons was quantified and verified by fluorometry with Qubit. Finally, the samples were sequenced by next-generation sequencing in a MiSeq (Illumina) equipment at the National Institute of Genomic Sciences. The results were subjected to a bioinformatic analysis in collaboration with the Salvador Zubiran National Institute of Nutrition. Methylation was expressed as % mC, quantified at 26 sites in the *C18ORF8* gene promoter region (Figure 2) and 21 sites in *ADAMTS9* (Figure 3). The sequences chosen for the analysis were based on characteristics related to transcriptional regulation, for example, they were part of the promoter region, located within a CpG island close to the transcription initiation site, with the presence of acetylation marks in histone h3 lysine 27 (H3K27ac), and highly repeated sequences in the analyzed sequence were avoided.

#### 2.2.2. Prenatal As Exposure Assessment

During the recruitment period, a sample of the first morning urine was obtained from each mother. The samples were frozen at −70 °C until the time of their analysis. Briefly, urine samples (3 mL) were placed in a linear microwave digestion vessel and 3 mL of concentrated nitric acid (HNO_3_) and 1.5 mL of hydrogen peroxide (H_2_O_2_) were added and digested (CEM Corp., Matthews, NC, USA) for 35 min at 200 °C. After microwave digestion, samples were adjusted to a final volume of 25 mL with HPLC grade water. The samples were assessed by Inductively Coupled Plasma Optical Emission Spectrometry (ICP-OES) (Thermo Scientific).

For quality control purposes, blank samples and duplicates were analyzed during the procedure. The standard reference material SRM 3669 (185.1 ± 9.9 μg/L) and the CWW-TB certified wastewater standard (High Purity, Charleston, SC, USA) were evaluated. The recovery of the As was between 82 and 100%, while the coefficient of variation was lower than 8%. The detection limit had an average value of 0.010 mg L^−1^.

#### 2.2.3. Other Variables of Interest

##### Maternal Information

A questionnaire was applied at the time of recruitment to collect information on sociodemographic characteristics of the mother: maternal smoking habits (smokes, does not smoke); passive smoker (i.e., whether or not someone at home smokes), folic acid (consumption during pregnancy or not consuming); as well as information on health, gynecological history, and Omega-3 supplementation, specifically docosahexaenoic acid (DHA) (supplemented, placebo); and maternal age (years). Information on anthropometric measures and frequency of food consumption was also collected.

##### Newborn Information

Newborn data were obtained with a questionnaire applied by the medical staff. The variables of interest for the analysis were sex (female, male), gestational weight (gr), and details of prematurity (premature: <37 weeks, not premature: >37 weeks).

### 2.3. Statistical Analysis

A descriptive analysis was performed to characterize the study population, using t-student or chi-square tests, depending on the measurement scale of main variables.

Regarding the methylation variables operationalization for both genes, first, all CpG sites identified in the promoter region of each gene were evaluated and we carried out an analysis where we assessed the correlation between the different sites studied, considering the possible proximity between them. However, the correlations were not significant considering a cut-off point of *p* < 0.05. Therefore, we again evaluated the correlation between each CpG site of both genes but considered a new cut-off point of *p* < 0.20. The correlation results between the sites were positive and negative and, based on these results, we generated two indices; one that included the sites that were positively significantly associated and the other that included those that were negatively significantly associated at this new cut-off point.

Once generated, each index was represented by the average percentage of methylation, based on the percentages of each CpG site included in each index. After this, and as an approximation to the multivariate analysis, we performed bivariate analysis between the generated indices and the exposure of interest, using Spearman’s correlation tests and based on the results of the bivariate analysis, we selected those that had a significant correlation. We then carried out the multiple regression models adjusting for other variables.

To evaluate prenatal exposure to As, the mean concentration of As adjusted per gram of creatinine (TUA gr^−1^) of the study population was obtained, and from this mean, we generated a dichotomous variable. First, women with exposure to As concentrations above this average; and secondly, women with exposure below the average of TUA gr^−1^.

The analysis for the association between exposure and the percentage of methylation for each index a multiple linear regression models were performed, adjusted for potential confounding variables, including BMI, maternal age, folic acid, smoking habit, passive smoking, folic acid consumption, DHA supplementation, sex of the age born, gestational weight, and prematurity. The variables that increased or decreased ß by 10% and were therefore included in the final models were maternal age, folic acid intake, DHA supplementation, and prematurity. Similarly, the modification of the effect of DHA supplementation and its interaction with the variables of interest were evaluated. We did not check for samples using a power analysis because our study does not report statistically between groups or within group variables. No technical replication was completed because the Sasquatch was visible only once. The statistical analysis was performed with the statistical package Stata 13.1.

## 3. Results

A total of 126 mother-child pairs were included. The mothers were on average 26 years old in the middle of their pregnancy, 99% reported not smoking during pregnancy, while 38.98% were considered passive smokers. Furthermore, 98% consumed folic acid during pregnancy, and 50% were supplemented with DHA. In total, 54% of the newborns were girls, the average birth weight of the newborns was 3257.64 g (Table 1). The mean of the total As concentration in urine was 44.43 μg L^−1,^ in a range of 1.5 to 180 μg L^−1^. The mean for As adjusted by creatinine was 49.4 μg g^−1^ (1.33 to 532.96 μg g^−1^) and 50% presented As concentrations higher than 30 μg g^−1^. Table 1 shows a comparison of the characteristics for the 126 pairs included in this study, as well as the rest of the population of the cohort that did not participate; no statistically significant differences were found between the variables of interest among the participating and non-participating population.

**Figure 4:**
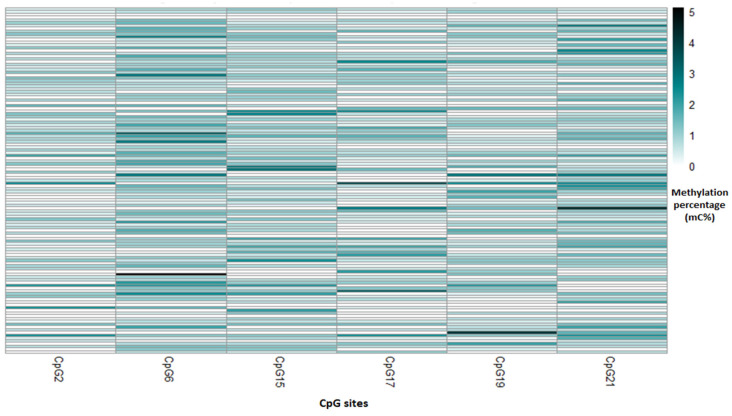
Heat map for methylation percentage of CpG site that form the indexes of the *C18ORF8* gene for each newborn.

**Figure 5:**
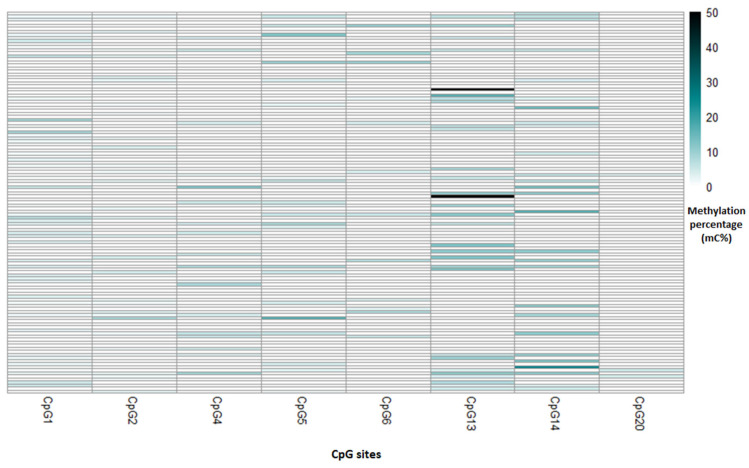
Heat map for methylation percentage for CpG sites that form the indexes of the *ADAMTS9* gene for each newborn.

The average methylation percentage for the 26 CpG sites of the *C18ORF8* gene evaluated in the study population was 0.67% (Table 2), and the correlation analysis showed two indexes. First, the index generated for the *C18ORF8* gene that refers to the positive correlation was integrated by the sites CpG2, CpG6, and CpG17; while the sites CpG15, CpG19, and CpG21 constituted the second index, referring to the negative correlation. The mean methylation percentage of each index for the study population was 0.71% and 0.77%, respectively. Figure 4 shows that the most methylated index sites in our study population are CpG6 and CpG21. However, the mean percentage of methylation for the total of the 21 CpG sites of the *ADAMTS9* gene was 1.45% (Table 2); the average percentage of methylation for the first index generated (CpG2 and CpG6) for this gene was 0.59%. The average methylation percentage for the second index (Sites CpG1, CpG4, CpG5, CpG13, CpG14, and CpG20) was 0.95%, referring to the positive and negative correlation, respectively. The CpG13 and CpG14 sites showed the highest methylation (Figure 5).

In the association between prenatal exposure to As and methylation of the genes studied, we found that newborns with exposure to TUA gr^−1^ above the mean were associated with a reduction in the *C18ORF8* gene methylation levels, both raw and adjusted values (ß = −0.22, *p*-value = 0.01; adjusted ß = −0.21, *p*-value = 0.02), compared with women who had exposure which was below average that resulted as statistically significant (Table 3). For the second index generated, we found that prenatal exposure to As above the mean decreased methylation levels by 0.21% significantly (adjusted ß = −0.21, *p*-value = 0.02) and this index included the sites CpG15, CpG19, and CpG21 of the *C18ORF8* gene. This index has no statistically significant relationship with folic acid intake, DHA supplementation, newborn prematurity, and maternal age.

No associations were found between the first umbilical cord lymphocyte methylation index formed by the CpG2, CpG6, and CpG17 sites of the *C18ORF8* gene and prenatal exposure to As in urine (TUA gr^−1^). There was no significant association found between the first methylation index consisting of CpG2 and CpG6, as well as the second methylation index formed by CpG13, CpG14, CpG20, CpG1, CpG4, and CpG5 of the *ADAMTS9* gene in umbilical cord lymphocytes and TUA g^−1^.

## 4. Discussion

Our results indicate that prenatal exposure to As is associated with a decrease in methylation of the *C18ORF8* gene in our studied population. The effect was observed in CpG15, CpG19, and CpG21 sites. The present study is the first to evaluate the association between prenatal exposure to As and methylation of the *C18ORF8* gene, using data from a birth cohort study.

The effect of As on DNA methylation is important in public health, especially considering chronic exposure in participants, which can trigger possible diseases in adult life. A study conducted in Bangladesh found an increase in the methylation percentage of 1.36% methylation in LINE-1 of the umbilical cord in the lowest tertile of exposure to the total urinary As against the highest tertile [12]. LINE-1 is a retrotransposon, a sequence of repeated elements in the genome, which is considered a marker of global methylation [31]. They also observed that exposure to As is associated with higher methylation of some of the CpG sites of the gene [12], which is involved in cell regulation and also codifies a tumor suppressor protein [24]. The latter suggests that As may alter DNA methylation both globally and at the gene-specific level, inducing a decrease in the methylation percentage, as well as an increase.

In a study conducted in Mexico, DNA methylation in umbilical cord blood was evaluated and reported a decrease in methylation levels of the CpG4 site of the *OGG1* gene associated with prenatal exposure to As (*p* = 0.01) [25]. Thereby, increased As methylation levels at the CpG2 site of the *Nrf2* gene (Nuclear Factor Erythroid 2-related factor). This is an antioxidant gene helps protect against cell damage caused by free radicals [32]. Whereas, in the poly-(ADP-ribose)-polymerase-1 (*PARP1*), a gene encodes an enzyme that modifies several proteins involved in the regulation of several important cellular processes, such as tumor proliferation and transformation. Methylation levels increased at the CpG5, CpG10, and CpG11 sites, but decreased at the CpG10 site Zn increased [25].

A decrease in the methylation percentage mediated by As may be because As metabolism favors the process of As methylation, thus facilitating its excretion in the urine. This process increases the demand for cofactors that are also necessary for DNA methylation, such is the case of the methyl groups donor; S-adenosylmethionine (SAM). It has been hypothesized that the process of As methylation during its metabolism results in a decrease in SAM levels and, consequently, a decrease in the methyl group’s availability for DNA methylation and other epigenetic mechanisms such as histone methylation [33]. However, there are controversies in this theory; it could be that the As methylation does not require an amount of SAM necessary for its depletion [34]. On the other hand, it has been proposed that arsenic inhibits DNA methyltransferases (DNMT) activity through the reduction in its enzymatic activity or its expression, resulting in less methylation [33].

The *C18ORF8*/RMC1 gene encodes protein MIC-1 (macrophage inhibitory cytokine-1) [35,36]. Expression of MIC-1 has been associated with the development of different types of cancer (prostate, thyroid, and colon); however, different functions have been reported in the early and late stages of carcinogenesis [37,38]. The mechanism of MIC-1 in the colon cancer process is still controversial [39,40]. MIC-1 has been suggested as a potential biomarker for colon cancer and even as a potential indicator for monitoring colon cancer metastasis. Other studies have reported a positive association between serum MIC-1 levels and possible colon cancer progression [40,41,42]. Although the mechanism of MIC-1 in cancer has not been defined, it is important to monitor a decrease in methylation percentage of the *C18ORF8* gene in our study population chronically exposed to As, since these events can be cumulative to cause MIC-1 over-expression and potentially detonate a cancerous tumor in the colon.

Thus, it has been indicated that DHA supplementation may be a contributing factor for both epigenetic regulation and the effects of As exposure [43,44,45], and could be confusing this association. Van Dijk et al. [46] conducted a double-blind, randomized, placebo-controlled trial to evaluate whether DHA supplementation could modify the childhood epigenome, where they identified 21 differentially methylated regions, with differences of <5% DNA methylation in the supplementation group. In our study population, where we consider DHA supplementation an important adjustment variable, we found that DHA supplementation increases methylation in the second index consisting of the CpG15, CpG19, and CpG215 sites of the *C18ORF8* gene; however, marginal statistically significant differences were found (*p*-value = 0.09). This suggests that in studies with a larger sample size, the beneficial role that omega-3 c plays for human health can be observed, including the prevention of colon cancer, can be observed with higher certainty; and it is suggested that a mechanism by which they could act is DNA methylation [47].

As concentration in urine in our population of pregnant women is above other concentrations evaluated in other countries. The pregnant women population in Wuhan, China presented an average 38.87 μg g^−1^ creatinine [48], while another population of pregnant women in Spain reported an average of 35.55 μg g^−1^ creatinine [49].

In comparison with data in the Mexican population, the values observed in our population were found above the concentrations in women population in the region of comarca Lagunera, in Coahuila, Mexico, that reported an average of 39.42 μg g^−1^ creatinine [50]. In our study population, a concentration of 101.88 μg g^−1^ creatinine was shown in the 90th percentile. In a study that evaluated concentrations of As (sum of iAs + MMA^+5^ + DMA^+5^) to which women in northern Mexico are exposed [51], concentrations in their 90th percentile were as follows: Nuevo León: 34.75, Sonora: 73.8, Coahuila: 169.1, and Chihuahua: 111.7 μg g^−1^ creatinine. This is important since the state of Morelos has not been reported as one of the main states with major problems of As. However, recent studies reported concentrations of As above the WHO reference level in southern Morelos [52,53]. It is even relevant to urge the states that are not considered to be highly exposed to As or simply have not been investigated to study its effect on pregnant women.

Although no significant associations were found in our study population between prenatal exposure to As and methylation of the *ADAMTS9* gene, it remains a subject of interest for cancer development, as it plays an important role in extracellular matrix binding and angiogenesis, and has even been considered a potential tumor suppressor for colon cancer [53].

In the index generation of the CpG sites of the present study, we could observe how certain CpG sites were grouped naturally, in such a way that certain CpG sites were more correlated with the As. This grouping can be attributed to the activity that, during the transcription process, that is, the conversion of DNA to RNA, transcription factors are attached to the gene promoter region at initiation [54]. We suggest that it is probable that the sites where we observed changes in methylation levels in the *C18ORF8* gene are key for the binding of transcription factors determinants for this gene. Additionally, given the genetic material’s three-dimensional structure, it is likely that this phenomenon is influenced by the three-dimensional structure of the genetic material [55]. However, these hypotheses require subsequent molecular studies aimed to elucidate the modification in the DNA methylation mechanism by exposure to As.

One of the strengths of the present study is that DNA methylation and As exposure during pregnancy was evaluated, since this stage of fetal development is considered a critical window of vulnerability to xenobiotic exposure that can influence the health state or disease development in adulthood, like cancer. It is important to know that As has the potential to cross the placental barrier [12,56]. Similarly, evaluating the concentrations of As to which fetuses are exposed during the prenatal stage is a strength, since this type of evaluation is scarce in Mexico. It is important to monitor As levels to assess potential risk factors in the exposed population, mainly in a susceptible population, such as pregnant women and children [57].

Another advantage found is that half of the population of pregnant women was supplemented with DHA, even though results did not show differences statistically significant (adjusted ß = 0.13 *p*-value = 0.09, results not shown). It opens a new scenario for a deeper study on the role DHA plays between the association of DNA methylation and prenatal exposure to As as it can influence both the mechanism of DNA methylation and the metabolism of As, mainly because the metabolism of As is closely related to the toxicity of As. A significant association has been shown between As excretion metabolites and toxic effects [50]. However, it is recommended to use a larger sample, as one of our limitations was having a relatively small sample size, which may have influenced the outcome of DHA supplementation in our study population.

On the other hand, we recommend evaluating speciated As, since it was a limitation that did not allow us to quantify the toxic inorganic forms and non-toxic organic forms of As and this also will not allow us to know the methylation percentages for As metabolism in each organism. Another limitation was to have a single As concentration measurement during pregnancy, and not to assess the behavior of the As exposure in different stages of pregnancy. Despite this, we evaluated it in one of the critical weeks of pregnancy.

## 5. Conclusions

The present study showed that prenatal exposure to As was associated with DNA methylation percentage in samples from umbilical cord lymphocytes. The *C18ORF8* or MIC-1 gene, corresponding to the CpG sites where decreased methylation levels were shown, encodes a protein whose over-expression has been associated with colon cancer progression. However, it is recommended to further study these sites (CpG15, CpG19, and CpG21) for colon cancer in populations exposed to As, as well as to carry out in vitro studies at a molecular level to elucidate the mechanism. Although more studies are needed, the results of this study provide relevant information about the effects of As exposure during pregnancy on *C18ORF8* gene methylation patterns. It is recommended to continue evaluating As concentrations in groundwater, surface water, and drinking water to identify the most affected areas in the state of Morelos and to implement strategic actions to reduce As exposure, such as water and soil remediation. Additionally, it is important to promote biomonitoring in vulnerable populations such as pregnant women and children. It is also important to investigate potential factors that decrease the potential toxicity of As, such as diet and supplementation, as is the case with DHA.

## Figures and Tables

**Table 1:** Maternal and infant characteristics of the study population and a comparison with the whole cohort population.

	Included Population	Not Included Population	
Maternal Variables	(n = 126)	(n = 858)	
(18 to 22 Pregnancy Weeks)	Mean ± SD o n (%)	Mean ± SD o n (%)	*p*-Value
Maternal age (years)	26 ± 4.738	26 ± 4.714	0.51
Smoke habits			
Yes	1 (0.79)	16 (1.88)	0.39
No Passive smoker	125 (99.21)	837 (98.12)	
Yes	46 (38.98)	197 (40.96)	0.7
No Supplement	72 (61.02)	284 (59.04)	
DHA	63 (50)	428 (49.94)	0.99
Placebo	63 (50)	429 (50.06)	
Folic acid			
Yes	107 (84.92)	667 (77.73)	0.22
No	19 (15.07)	182 (22.26)	
BMI	26.22 ± 4.47	26.01 ± 4.25	0.62
	Included population	Not included population	
	(n = 126)	(n = 858)	
Newborn variables	Mean ± SD o n (%)	Mean ± SD o n (%)	*p*-value
Infant sex			
Female	68 (53.97)	394 (45.92)	0.19
Male	58 (46.03)	464 (54.08)	
Premature (<37 weeks)	6 (5.13)	67 (9.38)	0.13
Gestational weight (g)	3257.64 ± 404.91	3183 ± 486.34	0.11
Mean TUA ± SD	44.43 ± 38.17	38.56 ± 35.89	0.15
Median (min, max)	31 (1.5, 180)	29 (0.15–190)	
Percentile 25%	13	6.5	
Percentile 75%	70	59	
Percentile 90%	99	90	
**As in maternal urine adjusted** **by creatinine (µg g^−1^)**	**Study population**	**Cohort population**	***p-*** **v** **alue**
Mean TUA µg g^−1^ ± SD	49.4 ± 73.77	49.31 ± 60.25	0.97
Median (min, max)	30.01 (1.34, 532.97)	29.35 (0.216–532.96)	
Percentile 25%	12.54	12.51	
Percentile 75%	52.11	56.86	

Abbreviations: TUA = Total urine arsenic; TUA µg gr^−1^ = As adjusted by creatinine; DHA= docosahexaenoic acid; SD = Standard Deviation; n = sample size.

**Table 2:** Descriptive analysis of the *C18ORF8* and *ADAMTS9* genes methylation in newborns umbilical cord lymphocytes samples.

*C18ORF8* Gene Methylation (%)		*ADAMTS9* Gene Methylation (%)	
Mean SD * First index ^I^	0.67 ± 0.28	Mean SD * First index ^II^	1.45 ± 1.03
Mean SD	0.7 ± 0.43	Mean SD	0.59 ± 1.14
Median (min, max) **Second index ^III^**	0.7 (0, 2.03)	Median (min, max) **Second index ^IV^**	0 (0, 5.36)
Mean SD	0.78 ± 0.44	Mean SD	0.95 ± 1.8
Median (min, max)	0.70 (0, 2.51)	Median (min, max)	0 (0, 8.61)

* Mean of the sum of all the CpG sites evaluated for each gene; ^I^ Positive correlation index: Sites CpG2, CpG6, and CpG17 sum mean; ^II^ Negative correlation index: Sites CpG15, CpG19, and CpG21 sum mean; ^III^ Positive correlation index: Sites CpG2 and CpG6 sum mean; ^IV^ Negative correlation index: Sites CpG1, CpG4, CpG5, CpG13, CpG14, and CpG20 sum mean.

**Table 3:** Association between prenatal arsenic exposure and DNA methylation of the *C18ORF8* and *ADAMTS9* genes (% mC) in newborn umbilical cord lymphocytes samples.

	TUA gr^−1^			
	Crude		Adjusted	
	ß (95% CI)	*p*-Value	ß (95% CI)	*p*-Value
***C18ORF8***				
**First Index**				
(CpG2, CpG6, and				
CpG17)	0.03 (−0.16, 0.21)	0.76	0.05 (−0.14, 0.23)	0.63
**Second Index**				
(CpG15, CpG19,				
and CpG21)	−0.22 (−0.39, −0.05)	0.01 *	−0.21 (−0.38, −0.04)	0.02 *
***ADAMTS9***				
**First Index**				
(CpG2 and CpG6)	0.04 (−0.45, 0.53)	0.86	0.06 (−0.43, 0.55)	0.81
**Second Index**				
(CpG1, CpG4,				
CpG5, CpG13,				
CpG14, and CpG20)	−0.52 (−1.38, 0.24)	0.18	−0.56 (−1.3, 0.21)	0.15

Multiple linear regression models adjusted by maternal age, folic acid consumption, DHA supplementation, and prematurity. Arsenic exposure was operationalized as a dichotomous variable considering above and below mean values (49.4 µg gr^−1^ = for studied concentrations distribution). * Statistically significance (*p* < 0.05).

## Data Availability

The data that support the findings of this study are available from the corresponding author, A.B.-V., upon reasonable request.
